# Duodenal Varices Presenting as Lower Gastrointestinal Bleeding

**DOI:** 10.7759/cureus.63244

**Published:** 2024-06-26

**Authors:** Olachi Egbo, Bassel Dakkak, Onyinye S Ugonabo, Christopher Magiera

**Affiliations:** 1 Department of Internal Medicine, Aurora Medical Center Oshkosh, Oshkosh, USA; 2 Department of Internal Medicine, Marshall University Joan C. Edwards School of Medicine, Huntington, USA; 3 Department of Gastroenterology, Aurora Medical Center Oshkosh, Oshkosh, USA

**Keywords:** ectopic varices, acute gi bleed, diagnosis of ectopic varices, lower gastrointestinal bleeding, duodenal varices

## Abstract

Duodenal varices pose a significant clinical challenge due to their association with severe gastrointestinal bleeding. This condition requires attention because of its acute severity, high morbidity, and mortality rates. The presented case underscores the importance of advancing both knowledge and treatment approaches for duodenal varices. This pursuit is aimed not only at improving immediate clinical outcomes but also at deepening our understanding of complications related to portal hypertension.

## Introduction

Portal hypertension occurs when the portal venous pressure abnormally increases, subsequently causing collateral connections between the portal and systemic circulations, primarily resulting in visibly enlarged veins called varices [1]. Ectopic varices are characterized as enlarged portosystemic collateral veins situated in atypical locations outside the gastroesophageal area [2]. Duodenal varices are ectopic varices that make up 1-3% of all varices found in patients with cirrhosis. While this proportion may appear modest, the notable aspect is the high mortality rate associated with duodenal varices, which can escalate to as much as 40% [3]. This condition was initially reported by Alberti in 1931 [4]. Duodenal varices arise primarily from chronic liver diseases such as cirrhosis and commonly occur in the duodenal bulb and the second portion of the duodenum [5]. The treatment approach includes a combination of endoscopic therapy, radiological intervention, and surgery [5]. Our case focuses on a patient with liver cirrhosis experiencing duodenal variceal bleeding manifesting as lower gastrointestinal bleeding, which was effectively managed with sclerosing therapy and a transjugular intrahepatic portosystemic shunt (TIPS).

## Case presentation

A 53-year-old male patient presented with painless bright red rectal bleeding and dizziness a few hours before admission. He has a known history of decompensated alcoholic cirrhosis but was lost to follow-up. Other medical history includes hypertension and obstructive sleep apnea on CPAP therapy. His alcohol history was significant for the consumption of approximately 12 fl oz of hard seltzer drinks daily, with his last drink being a day before presentation. He denied any hematemesis, coffee-ground emesis, or previous gastrointestinal hemorrhage. His first esophagogastroduodenoscopy (EGD) in 2014 showed no varices. His home medications include amlodipine 10 mg, metoprolol succinate 100 mg, furosemide 40 mg, and spironolactone 100 mg. Vitals were significant for sinus tachycardia with a heart rate of 124 bpm and borderline low blood pressure at 104/64 mmHg. On examination, there was bright red rectal bleeding. Significant laboratory results showed a decrease in hemoglobin levels, elevated blood ethanol, lactic acid, and liver enzymes (see Table 1). Computed tomography (CT) angiography of the chest, abdomen, and pelvis showed hepatic steatosis, signs of portal hypertension, and possible right portal vein stenosis or thrombosis. He was started on a proton pump inhibitor (PPI), an octreotide infusion, and transfused one unit of packed red blood cells.

**Table 1:** Significant laboratory results

Test Name	Value	Reference Range
White blood cell count	4.1	4.5–10 × 10^9^ cells/L
Hemoglobin	11.8	14–17 g/dL
Blood urea nitrogen	7	7–20 mg/dL
Creatinine	0.67	0.6–1.3 mg/dL
Prothrombin time (PT)	13	11–13 seconds
International normalized ratio (INR)	1.3	0.8–1.2
Lactic acid	3.8	0.5–2.2 mmol/L
Alanine transaminase (ALT)	94	7–56 IU/L
Aspartate transaminase (AST)	314	10–40 IU/L
Alkaline phosphatase	140	30–130 IU/L
Bilirubin (total)	2.8	0.1–1.2 mg/dL
Ethanol	154	0–10 mg/dL

**Figure 1:**
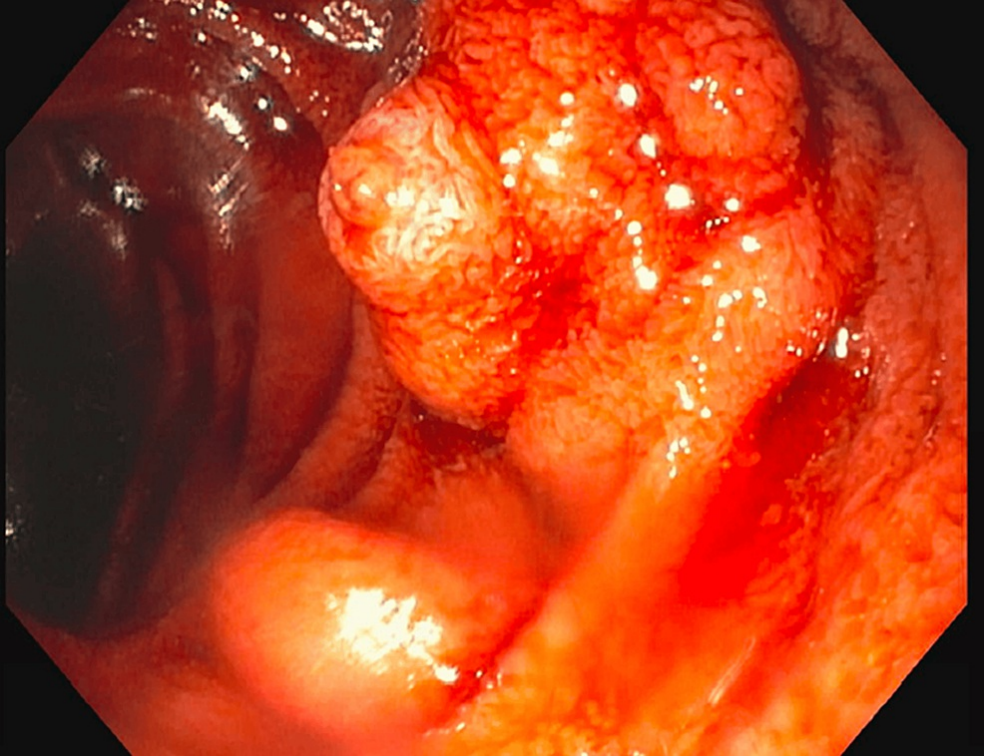
EGD findings of bleeding duodenal varices. EGD: esophagogastroduodenoscopy.

An EGD revealed moderate portal hypertensive gastropathy, small esophageal varices without stigmata, and actively bleeding duodenal varices (see Figure 1). The bleeding was successfully controlled with sclerosing therapy. To reduce the risk of rebleeding, the patient also had a TIPS and balloon-occluded retrograde transvenous obliteration (BRTO). He was discharged after four days in stable condition, and his home medications were resumed. Two weeks post-discharge, he attended his follow-up appointment and reported no recurrence of rectal bleeding.

## Discussion

Gastrointestinal (GI) bleeding is divided into upper and lower bleeding. Lower GI bleeding is defined as bleeding that starts below the ligament of Treitz and usually presents as bright red blood per rectum. Bleeding above the ligament of Treitz usually presents as melena or hematemesis. However, if the bleeding is brisk, it may present as hematochezia [6]. Duodenal varices can be due to extrahepatic or intrahepatic causes. While liver cirrhosis accounts for the most common intrahepatic cause, extrahepatic causes can include portal hypertension, thrombosis, obstructive tumors, arteriovenous fistula, and pancreatitis [5, 7, 8]. Sadum et al. reported a case of duodenal variceal hemorrhage after a sleeve gastrectomy [9]. 

Duodenal varices represent a challenging clinical entity, as identifying these lesions as the origin of bleeding can be difficult. They are relatively rare compared to esophageal varices, and their uncommon location in the duodenum makes them less likely to be considered. Establishing a diagnosis may require multiple successive endoscopies (EGDs) [10]. Unlike esophageal varices, duodenal varices are not frequently screened for, leading to potential delays in diagnosis and management. Duodenal varices present with rare but fatal gastrointestinal bleeding. The diagnosis of duodenal varices is predominantly confirmed through endoscopy [11]. CT and magnetic resonance imaging are useful for visualizing the portal venous system and assessing the extent of liver disease. Doppler ultrasound is also beneficial for evaluating blood flow in the portal vein and detecting varices. Finally, capsule endoscopy is a less invasive technique that can be used in patients where conventional endoscopy is not feasible or inconclusive [11].

The treatment options for duodenal varices can be divided into endoscopic, interventional radiology procedures, and surgery. Endoscopic therapeutic modalities include endoscopic band ligation, endoscopic injection sclerotherapy, endoscopic tissue adhesive (ETA), and combination therapy. Singh et al. reported ETA treatment with cyanoacrylate glue that resulted in successful obliteration of the duodenal varices [12]. While the injection of cyanoacrylate is considered a viable endoscopic treatment for bleeding duodenal varices, there are technical challenges associated with glue injection, such as determining the optimal method for accessing the vessel when it is situated in the second part of the duodenum [13]. A meta-analysis by Yipeng et al. [14] reported an overall treatment success of 81.2% among patients who had endoscopic intervention. However, adverse events noted include endoscopic treatment-induced ulcer, portal vein thrombosis, biliary obstruction, pulmonary embolism, abdominal pain, and sepsis [14].

Compared to endoscopic therapy, interventional radiology procedures like TIPS decompress the portal system and are especially beneficial in cases of unsuccessful endoscopic treatment [15]. TIPS has its limitations, including shunt occlusion, a significant mortality rate, and hepatic encephalopathy [16]. BRTO assesses the portal vein retrogradely through the gastrorenal shunt and uses occlusion balloons to stagnate sclerosant material within the varix. It is used as a therapeutic adjunct or alternative to TIPS. Percutaneous transhepatic obliteration (PTO) works in a similar way to BRTO. Double balloon-occluded embolotherapy (DBOE) combines both BRTO and PTO and may be a better treatment option than BRTO [2]. DBOE approaches from both the feeding and draining veins, thus reducing the risk of bleeding compared to BRTO. Plug-assisted retrograde transvenous obliteration (PARTO) was introduced in 2013 by Gwon et al. [17]. It is also known as a modified BRTO and uses a vascular plug and gelatin sponge instead of an indwelling balloon catheter [18]. Though most reported cases of successful PARTO were in gastric varices, Lee et al. [19] reported a case of duodenal varices that was successfully treated with PARTO. This modified method, though having a shorter procedure time compared to BRTO, is incapable of achieving complete occlusion with a mesh material and cannot be applied to varices with a gastrorenal shunt exceeding 18 mm due to constraints in the size of the vascular plug [19]. Adjunct radiological interventions after endoscopic intervention depend on the clinical status of the patient and generally improve mortality.

Lastly, surgical procedures like duodenal resection and extrahepatic portosystemic shunts are performed in rare cases where endoscopic and interventional radiological treatments are not feasible or effective, such as in the case of extrahepatic portal venous obstruction [2, 7].

## Conclusions

In summary, duodenal varices are a challenging entity in diagnosis and treatment. They should be considered in the differential diagnosis for any patient presenting with lower gastrointestinal bleeding, alongside other causes of lower gastrointestinal bleeding. Diagnosing ectopic varices requires high clinical vigilance. Although non-invasive tests are available to detect ectopic varices, endoscopy remains the main method to confirm the diagnosis. Early detection is crucial to prevent life-threatening bleeding. The current choice of treatment modality depends on the clinical scenario and may include interventional radiology (TIPS and BRTO), combined with endoscopic methods. Further studies are needed to determine the best approach for treatment.
